# Long COVID: post-acute sequelae of COVID-19 with a cardiovascular focus

**DOI:** 10.1093/eurheartj/ehac031

**Published:** 2022-02-18

**Authors:** Betty Raman, David A. Bluemke, Thomas F. Lüscher, Stefan Neubauer

**Affiliations:** Division of Cardiovascular Medicine, Radcliffe Department of Medicine, Oxford Centre for Clinical Magnetic Resonance Research, University of Oxford, National Institute for Health Research (NIHR) Oxford Biomedical Research Centre (BRC), Oxford University Hospitals NHS Foundation Trust, John Radcliffe Hospital, Headley Way, Oxford OX3 9DU, UK; Department of Radiology, University of Wisconsin School of Medicine and Public Health, 3252 Clinical Science Center, 600 Highland Ave, Madison, WI 53792, USA; Department of Medical Physics, University of Wisconsin School of Medicine and Public Health, 3252 Clinical Science Center, 600 Highland Ave, Madison, WI 53792, USA; Royal Brompton & Harefield Hospitals and National Heart and Lung Institute, Imperial College, London, UK; Center for Molecular Cardiology, University of Zurich, Zurich, Switzerland; Division of Cardiovascular Medicine, Radcliffe Department of Medicine, Oxford Centre for Clinical Magnetic Resonance Research, University of Oxford, National Institute for Health Research (NIHR) Oxford Biomedical Research Centre (BRC), Oxford University Hospitals NHS Foundation Trust, John Radcliffe Hospital, Headley Way, Oxford OX3 9DU, UK

**Keywords:** COVID-19, Long COVID, Post-acute sequelae of COVID-19, Cardiovascular disease, Coronavirus, Long term

## Abstract

Emerging as a new epidemic, long COVID or post-acute sequelae of coronavirus disease 2019 (COVID-19), a condition characterized by the persistence of COVID-19 symptoms beyond 3 months, is anticipated to substantially alter the lives of millions of people globally. Cardiopulmonary symptoms including chest pain, shortness of breath, fatigue, and autonomic manifestations such as postural orthostatic tachycardia are common and associated with significant disability, heightened anxiety, and public awareness. A range of cardiovascular (CV) abnormalities has been reported among patients beyond the acute phase and include myocardial inflammation, myocardial infarction, right ventricular dysfunction, and arrhythmias. Pathophysiological mechanisms for delayed complications are still poorly understood, with a dissociation seen between ongoing symptoms and objective measures of cardiopulmonary health. COVID-19 is anticipated to alter the long-term trajectory of many chronic cardiac diseases which are abundant in those at risk of severe disease. In this review, we discuss the definition of long COVID and its epidemiology, with an emphasis on cardiopulmonary symptoms. We further review the pathophysiological mechanisms underlying acute and chronic CV injury, the range of post-acute CV sequelae, and impact of COVID-19 on multiorgan health. We propose a possible model for referral of post-COVID-19 patients to cardiac services and discuss future directions including research priorities and clinical trials that are currently underway to evaluate the efficacy of treatment strategies for long COVID and associated CV sequelae.

## Graphical Abstract

**Graphical Abstract:**
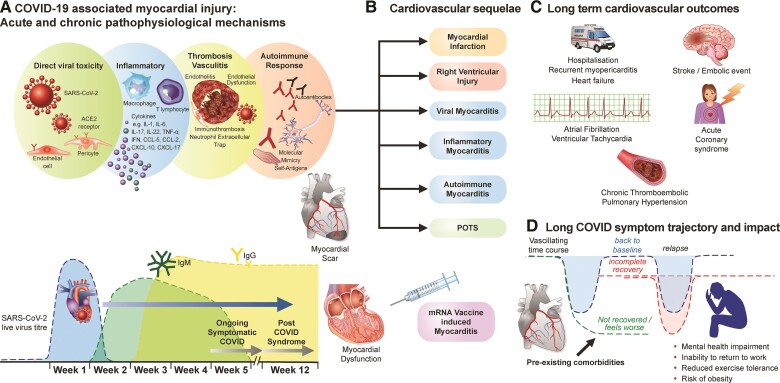
Central illustration depicting (*A*) Pathophysiological mechanisms underlying acute and chronic severe acute respiratory syndrome coronavirus 2-induced myocardial injury (including effects of vaccine) and its relation with timing of infection and onset of long COVID symptoms, (*B*) post-COVID-19 cardiovascular sequelae, (*C*) anticipated long-term cardiovascular complications and outcomes, and (*D*) the unpredictable trajectory of long COVID and its impact on mental health, ability to work, exercise tolerance, and potential to exacerbate the obesity epidemic. ACE2, angiotensin-converting enzyme 2; CCL, chemokine ligand; COVID, coronavirus disease; IL, interleukin; IFN, interferon; Ig, immunoglobulin; PCR, polymerase chain reaction; POTS, postural orthostatic tachycardia syndrome; RNA, ribonucleic acid; TNF, tumour necrosis factor.

## Background

The crippling effect of coronavirus disease 2019 (COVID-19) on healthcare and economies globally has undoubtedly been one of the worst disasters experienced by humans in the last decades. Worldwide, survivors of COVID-19 now exceed hundreds of millions,^1^ with some reporting incomplete recovery months beyond the acute illness, a condition commonly referred to as long COVID. Persistent symptoms of breathlessness, chest pain, fatigue, headaches, brain fog, and palpitations are a constant reminder of the devastation caused by this virus and the need to remain vigilant for any long-term damage. Now, more than ever before, management of cardiometabolic risk factors should become a priority for physicians, as their formidable power in intensifying COVID-19 illness severity has been convincingly documented. The long-term impact of COVID-19 on cardiovascular (CV) health and mortality is also emerging as a major global concern.

In this review, we discuss the definition of long COVID, epidemiology of cardiopulmonary manifestations in the context of long COVID, pathophysiological mechanisms for acute and chronic cardiac injury secondary to severe acute respiratory syndrome coronavirus 2 (SARS-CoV-2) infection, its management, and future directions.

## Long COVID and epidemiology of cardiac symptoms

The term ‘long COVID’ was originally coined by a patient^2,3^ and asserts the notion that suffering does not stop with resolution of acute infection. While there is no universally accepted definition, in December 2020 the United Kingdom (UK) National Institute for Health and Care Excellence guidelines^4^ defined long COVID as persistence of symptoms beyond 4 weeks of SARS-CoV-2 infection. This term comprises two phases: ongoing symptomatic phase (4–12 weeks) and post-COVID-19 syndrome (>12 weeks) based on the duration of symptoms. More recently, the World Health Organization provided a case definition for post-COVID-19 condition,^5^ a term used to refer to persistence of symptoms beyond 3 months of SARS-CoV-2 infection, lasting for at least 2 months and not explained by any other illness. Other terms used to describe long COVID include post-acute COVID-19 syndrome,^6^ post-acute sequelae of COVID-19,^7^ and long-haul COVID.^8^

**Figure 1:**
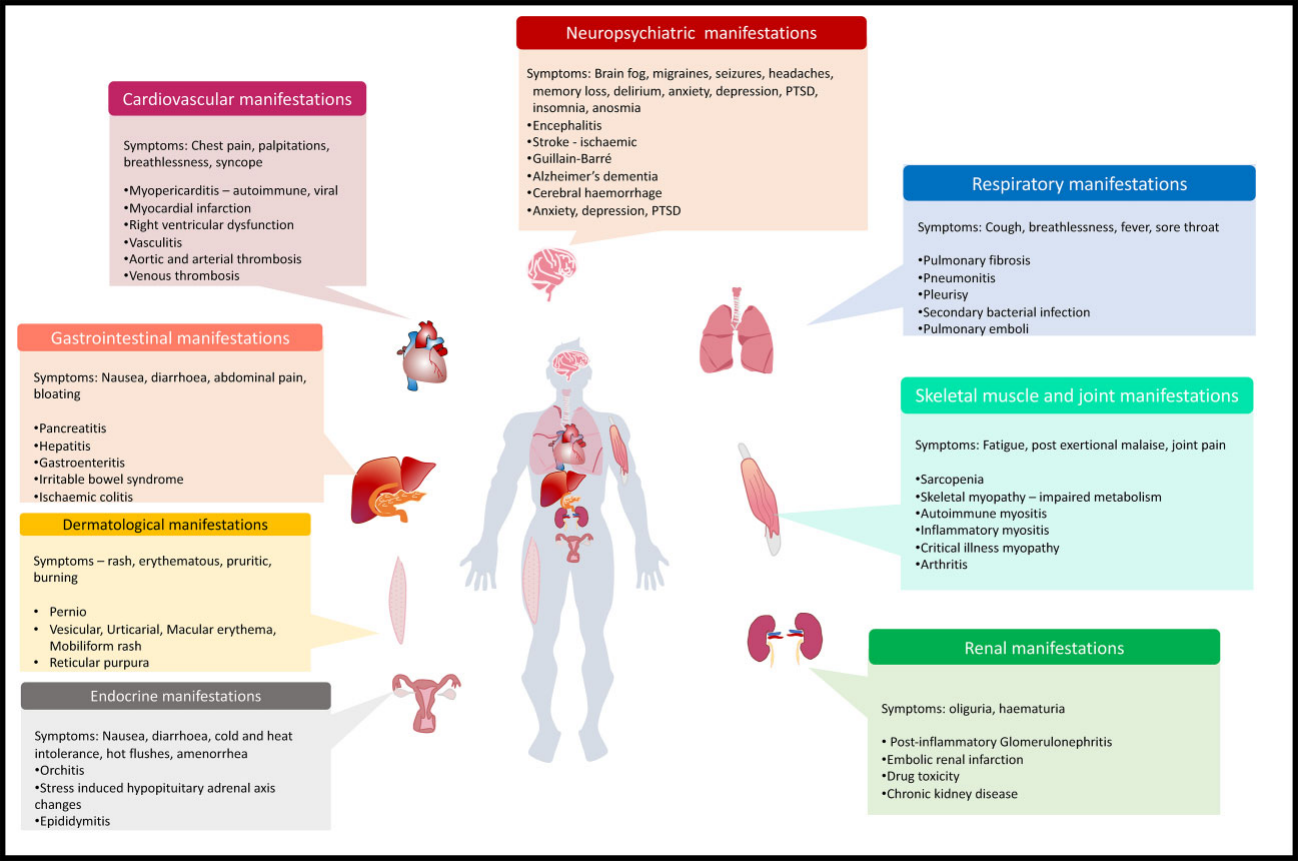
Long COVID is characterized by a diverse range of symptoms and signs spanning multiple organ systems including the respiratory system, neurological system, cardiovascular system, gastrointestinal system, dermatological system, endocrine/genitourinary systems, and skeletal muscle/joints as illustrated here. PTSD, post-traumatic stress disorder.

Long COVID is a vacillating disease,^9^ characterized by a diverse range of symptoms spanning multiple organ systems, as depicted in *Figure 1* and *Graphical Abstract*, and commonly includes fatigue, breathlessness, post-exertional malaise (PEM), brain fog, headaches, nausea, vomiting, anxiety, depression, skin rash, joint pain, and palpitations. Patient advocacy groups^6^ (e.g. long COVID SOS, COVID Advocacy Exchange, the National Patient Advocate Foundation COVID Care Resource Center, long-haul COVID fighters, Body Politic COVID-19 Support Group) have enhanced our understanding of this disease by drawing our attention to its multifaceted nature. Several experts^10,11^ have noted its marked similarities with other post-viral symptoms (e.g. Epstein-Barr,^12^ human herpesvirus,^13^ influenza, SARS,^14^ and Ebola viruses^15^), although few options exist for the management of such syndromes.

**Figure 2:**
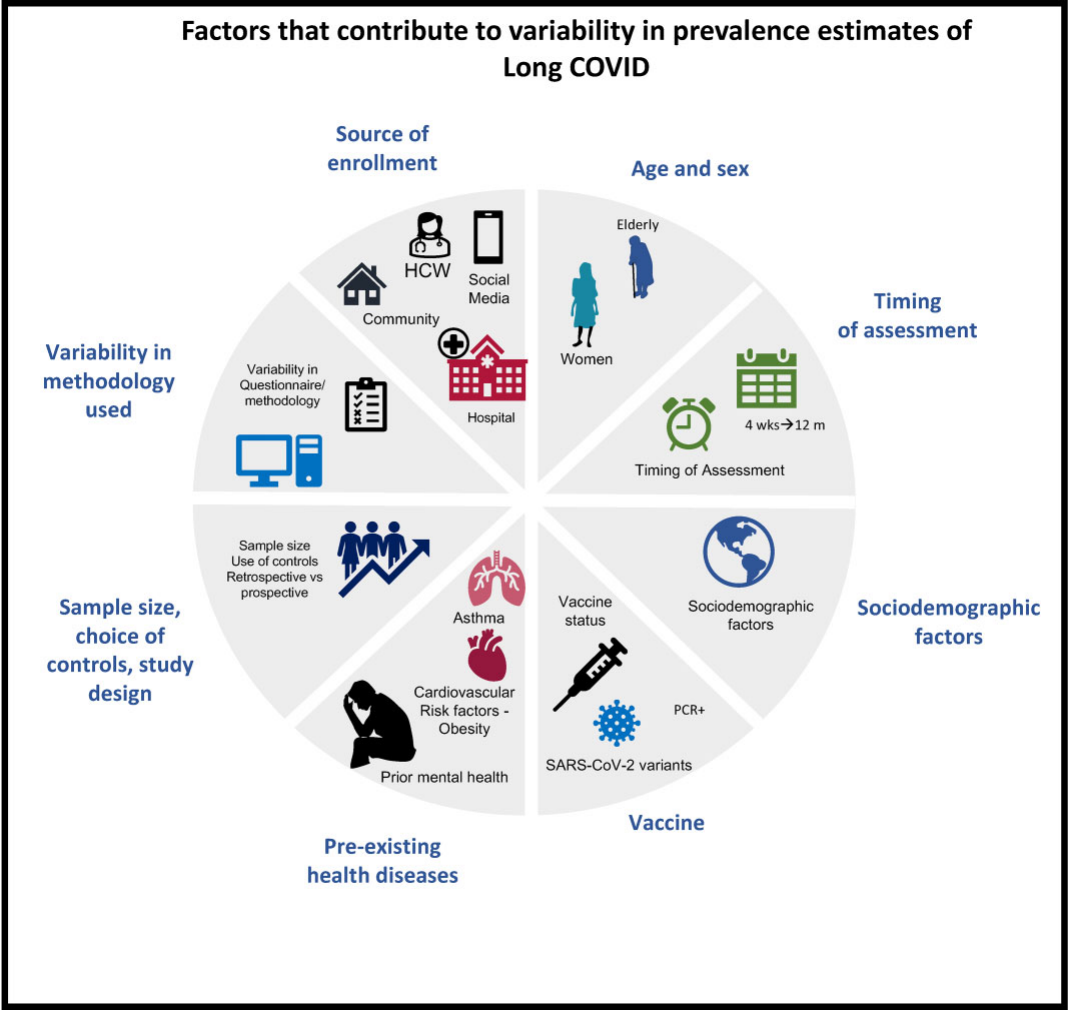
Factors that contribute to variability in prevalence estimates of long COVID. Prevalence estimates of long COVID are highly variable across studies due to a number of factors that introduce bias. These include differences in cohort characteristics, age, and sex of subjects enrolled, timing of assessment, sociodemographic factors, vaccines and variants, pre-existing health problems, sample size, study design, and variability in questionnaires or tools used. HCW, healthcare workers; m, months; PCR, polymerase chain reaction; wks, weeks.

The reported prevalence of long COVID has varied across and within many countries: UK 1.6–71%,^16–19^ Germany 35–77%,^20,21^ China 49–76%,^22,23^ Africa 68%,^24^ India 22%,^25,26^ Bangladesh 16–46%,^27,28^ Denmark 1%,^29^ Italy 5–51%,^30,31^ USA 16–53%,^32,33^ Norway 61%.^34^ Studies assessing hospitalized patients have typically reported higher prevalence estimates (e.g. 76% in Huang *et al*.,^22^ 71% in Evans *et al*.^19^) when compared with community studies (e.g. Sudre *et al*.^16^), reflecting the complex relationship between severity of acute illness, higher burden of co-morbidities, and persistent symptoms. Differences in the study population may explain the vast disparity in prevalence estimates across the various studies. The timing of assessment also appears to be important as symptom frequency can diminish over time from the infection. Hossain *et al.* ^28^ reported a reduction in the burden of long COVID symptoms which affected 21.2% of their cohort at 4 weeks and 16.5% by 12 weeks post-COVID diagnosis. A similar temporal improvement in symptom burden was also observed by Wu *et al*.^35^ and Cassar *et al*.^36^ Varying definitions of long COVID may also affect the relative frequencies. Mahmud *et al*.^27^ defined long COVID as the persistence of symptoms beyond 2 weeks (i.e. time taken for viral clearance) and reported a symptom prevalence of 46%. In contrast, more conservative definitions such as the one used by the UK Office of National Statistics (requiring the presence of functional limitation and exclusion of symptoms explained by co-morbidities) have resulted in lower prevalence estimates. The study design is also likely to be relevant as retrospective reporting from electronic healthcare records suffers from ascertainment bias, while prospective studies with comprehensive assessments^37^ are likely to attract patients with a high burden of symptoms seeking an explanation. In addition to these, disparities in vaccinations, SARS-CoV-2 variants, co-morbidities, study sample size, and use of varying non-COVID control groups appear to drive heterogeneity in prevalence estimates, as illustrated in *Figure 2*.

Contrary to the variability seen in reported disease prevalence, risk factors for long COVID tend to be fairly consistent, with female sex, escalating age, obesity, asthma, poor general health, poor pre-pandemic mental health, poor sociodemographic factors emerging as important determinants across several studies.^16–18,38–40^ In particular, the impact of nationwide lockdowns, remote working, and limited physical activity on pre-existing trends of an increasingly obese population with poor dietary intake and physical activity patterns is noteworthy.^41–43^ According to national statistics data from the UK (from 2019),^44^ among adults 16 and over, a staggering 68% of men and 60% of women were either overweight or obese. Obesity increased across all age groups up to 75 years old. In a separate report by the national child measurement programme from the UK, one in three children leaving primary school were noted to be overweight or obese and one in five obese in 2019.^45^ A recent update from the American Heart Association (AHA)^46^ on stroke and CV disease has also highlighted the high prevalence of obesity, metabolic syndrome, poor dietary habits, and physical inactivity among children and adults from the US. It is now well established that obesity and other cardiometabolic risk factors commonly promote inflammation and endothelial dysfunction,^47,48^ which may lower the cardiometabolic reserve and threshold for exertional symptoms. Consistent with this, numerous population and prospective cohort studies have documented an independent link between obesity and long COVID.^16,19,38^ Thompson *et al.*,^38^ in a prospective study of 6907 patients (mean age 19–63 years), reported that being overweight or obese was associated with a 25% higher likelihood of long COVID than not belonging to this category. Similarly, Sudre *et al.* ^16^ reported that patients with prolonged symptoms were more likely to be obese than those without.

Cardiopulmonary symptoms including chest pain, dyspnoea, fatigue, palpitations, and cough are common among long haulers. In one UK study,^16^ 13.3% of 4182 symptom app users (predominantly community patients) experienced at least one persistent symptom beyond 4 weeks of infection, of which half were thought to be cardiac in origin. In December 2020, the UK ONS^49^ provided similar estimates of long COVID prevalence, though a recent analysis with a stricter case definition (symptoms explained by co-morbidities were not counted) by the ONS^18,50^ and other groups^29^ suggests lower prevalence estimates (1.2–1.5%). Patient-led or survey-based research from long COVID support groups^37,51^ has also provided insights into the longitudinal trajectory of persistent symptoms. In an international online survey study undertaken by Davis *et al.* ^37^ of 3762 patients, cardiac symptoms including chest pain (∼53%), palpitations (∼68%), fainting (∼13%) were observed in up to ∼86% of patients by 7 months from infection. Postural orthostatic tachycardia syndrome (POTS), characterized by an increase in heart rate of at least 30 b.p.m. from supine to standing position, was noted in 31% of patients.^52^ Ziauddeen *et al.* ^51^ similarly studied the prevalence of long COVID among 2550 patients using a social media survey. Cardiopulmonary symptoms were reported by 89% of participants in their study.

There are now several prospective follow-up studies of hospitalized patients. One of the earliest published reports of long COVID came from an Italian study of hospitalized patients by Carfi *et al*.,^53^ demonstrating a particularly high burden of cardiopulmonary symptoms (>43%). In a subsequent follow-up study of 1733 hospitalized patients from Wuhan, China, Huang *et al.* ^22^ observed that at 6 months post-infection, 63% of patients reported fatigue, 26% breathlessness, and 5–9% experienced chest pain and palpitations. By 12 months,^23^ investigators of the same study showed that symptoms of breathlessness (30%) and chest pain (7%) were slightly more common, while fatigue had improved (20%). Evans *et al.* ^19^ from the UK also undertook a follow-up study of 1077 hospitalized patients. At a median of 5 months post-discharge, 48% reported persistent fatigue, 41% dyspnoea, and 21–28% chest pain and palpitations.

Long COVID has been proposed to be a form of chronic fatigue syndrome (CFS)/myalgic encephalitis.^52^ While there are marked similarities between the two, subtle yet important differences exist. Central to the diagnosis of CFS is the observation that patients experience PEM,^54,55^ defined as fatigue following even minor physical or mental exertion. For a diagnosis of CFS, symptoms should last for a minimum of 6 months and occur at least 50% of the time. Current definitions of long COVID do not obligate the presence of PEM, with symptom duration yet to be defined. A further point to note is that, while the evidence for exercise rehabilitation in CFS patients is mixed, early positive data supporting tailored rehabilitation in previously hospitalized patients with long COVID are emerging with some improvement seen in exercise capacity and cognition at 4 months from discharge.^56^ Finally, breathlessness, one of the most common long COVID symptom, is not essential for the diagnosis of CFS and may be due to distinct mechanisms. Nonetheless, the similarities between CFS and long COVID are striking, with both syndromes having links to psychological and neurostructural/metabolic alterations^57–59^ (e.g. grey matter volume reduction in the limbic cortex in post-COVID patients^60^), highlighting the potential benefits of neuroprotective interventions in such patients.

## Possible mechanisms underlying cardiac manifestations

Given the abundance of cardiac symptoms among patients with long COVID, a deeper discussion of potential mechanisms underlying cardiac injury is warranted.

### Acute phase

The role of angiotensin-converting enzyme 2 receptors in SARS-CoV-2 involvement of the heart is now well established. Several mechanisms have been proposed to contribute to myocardial injury including direct cytotoxic injury,^1^ dysregulation of renin–angiotensin–aldosterone system,^3^ endotheliitis and thromboinflammation, and^4^ dysregulated immune response with cytokine release.^6^

#### Autopsy studies

The pattern of myocardial injury following SARS-CoV-2 infection derived from autopsy studies suffers from referral bias but has provided initial pathophysiological clues. In an early autopsy series of 80 consecutive SARS-CoV-2 PCR positive cases,^61^ only four patients (5%) had suspected cardiac injury. Two patients had co-morbid conditions and died of sudden cardiac death. One had acute myocardial infarction and another exhibited right ventricular lymphocytic infiltrates. These early results suggested that extensive myocardial injury as a primary cause of death may be infrequent.

In a subsequent multicentre autopsy study, Basso *et al.* ^62^ characterized the hearts in 21 selected autopsies. Myocarditis (defined as lymphocytic infiltration and myocyte necrosis) was evident in 14% of cases, interstitial macrophage infiltration in 86%, and pericarditis and right ventricular injury in 19%, respectively. Halushka and Vander Heide^63^ performed a review of 22 publications describing the autopsy results of 277 patients. Lymphocytic myocarditis was reported in 7.2%; however, only 1.4% met the well-established histological criteria^64^ for myocarditis, suggesting that true myocarditis was rare.^65–67^ In another study, Lindner *et al*.^68^ demonstrated the presence of SARS-CoV-2 viral particles in the hearts of 24/39 (59%) consecutive autopsies; the viral load was clinically relevant in 16/39 (41%). Of note, viral particles were not isolated within cardiomyocytes, but rather in interstitial cells including pericytes and macrophages. The high viral load in some cases was also not associated with inflammation, consistent with the low prevalence of myocarditis on autopsy studies.^67^

#### Microvascular injury due to SARS-CoV-2

Cardiac troponin levels are frequently elevated in COVID-19 patients,^69^ indicating myocardial injury and/or ischaemia. The work of Bois *et al*.^70^ appears to support the concept of microthrombi occurring in association with COVID-19. In a small series of 15 individuals, the authors observed that post-mortem fibrin microthrombi were more common (80%) than acute ischaemic injury (13%) and myocarditis (33%) suggesting a role for thrombosis in accentuating myocardial injury.

Fox and Vander Heide^65^ have conceptualized the array of pathophysiological mechanisms underlying myocardial injury. The authors proposed that hypoxia and pulmonary microvascular damage may lead to right heart stress and myocyte necrosis. The latter may be further augmented through localized microvascular effects, endotheliitis,^71^ associated microthrombi, and altered renin–angiotensin homeostasis.^72^ Elevated cytokines^73,74^ [e.g. interleukins (IL)-1, IL-16, IL-17, IL-22, interferon (IFN)-γ, tumour necrosis factor (TNF)-α] could also contribute to myocardial injury by inducing endothelial dysfunction, activation of platelets, recruitment of neutrophils, and eventually triggering a hypercoagulable state. In this framework, viral myocarditis would play an infrequent role in SARS-CoV-2 infection.

### Post-acute and chronic phase

Mechanisms for enduring cardiac damage post-acute illness are still poorly understood. One possible explanation is a chronic inflammatory response evoked by persistent viral reservoirs in the heart following the acute infection,^75^ which may, in turn, be exacerbated by obesity-related inflammatory signalling driven in part by perivascular adipose tissue via the release of adipokines such as monocyte chemoattractant protein-1 and Regulated upon Activation, Normal T Cell Expressed, and Presumably Secreted, chemokines that aggravate endothelial dysfunction via endothelial nitric oxide synthetase uncoupling and reactive oxygen species production.^76^ An unintended consequence of such processes would be insidious tissue damage, followed by chronic myocardial fibrosis leading to impaired ventricular compliance, impaired myocardial perfusion, increased myocardial stiffness, reduced contractility and potential arrhythmias.

A second mechanism for delayed damage is an autoimmune response to cardiac antigens through molecular mimicry.^77^ High-throughput proteome analysis by Wang and others^78–80^ has identified a range of autoantibodies to humoral and tissue antigens in patients with severe COVID-19. Autoantibodies to cholinergic and adrenergic receptors have also been detected in individuals with CFS.^81,82^ Recently, a number of longitudinal cytokine profiling and proteomic studies^83,84^ have revealed an increased expression of prothrombotic factors (e.g. factor VIII, prothrombin, plasminogen activator inhibitor-1) beyond the acute infection. This is in keeping with the burgeoning reports of delayed embolic complications.^85–87^ The high prevalence of pulmonary vascular thrombosis (5–30%),^87,88^ particularly in hospitalized patients, is also expected to heighten the future risk of chronic thrombo-embolic pulmonary hypertension.^89^ Endothelial dysfunction^90^ and its complications may also develop in patients, with evidence of persistent impairment detected in younger individuals 3–4 weeks after SARS-CoV-2 infection.^91^ The *Graphical Abstract* summarizes the relevant pathophysiological mechanisms for acute and chronic cardiac injury secondary to SARS-CoV-2 infection and potential long-term consequences. It is worth noting that many of these complications mirror those encountered by survivors of other epidemics caused by SARS, Middle East respiratory syndrome (MERS), H1N1A, underscoring the need to recognize the impact of respiratory viral infections on CV health as previously outlined by Xiong *et al.* ^92^ in an earlier review.

## Post-acute COVID-19 cardiovascular sequelae

The high mortality and poor outcomes associated with myocardial injury^93–97^ during acute COVID-19 infection have galvanized an interest among research communities to characterize the long-term CV effects of SARS-CoV-2 infection. Insights from both prospective and retrospective studies continue to shape our understanding of its long-term effects. While retrospective studies rely on electronic medical health records and labelled datasets,^7,34,98^ prospective studies^19,22^ have innovatively turned to remote (e.g. telemedicine, symptom apps) assessments and face–face reviews.

### Retrospective cohort studies

There is now compelling evidence from large retrospective cohort studies that speak to the rising cases of new cardiac diagnoses. In a study of 73 435 (median age 61 years, 88% men) non-hospitalized patients using the US Department of Veterans Affairs health services, Al-Aly *et al.* ^7^ demonstrated a high risk of death and incident CV and metabolic diseases associated with COVID-19 beyond 30 days of infection. A UK-based study of 47 780 hospitalized COVID-19 patients (mean age 65 years, 55% men) demonstrated that a diagnosis of COVID-19 was linked to a three-fold increased risk of major adverse CV events up to 4 months from diagnosis (vs. non-hospitalized controls). In this study, Ayoubkhani *et al.* ^98^ further noted that the increased risk was not confined to the older age group and was more pronounced in non-White patients. Daugherty *et al.*,^99^ in a related paper, compared the incidence of new cardiometabolic diagnoses in post-COVID-19 patients with two important controls groups—non-COVID-19 controls (from 2019 to 2020) and those recovering from lower respiratory tract infection (LRTI). In their study, COVID-19 was associated with a nearly two-fold increased risk of incident CV diagnoses. However, when comparisons were made with LRTI controls, the excess risk of cardiomyopathy was no longer significant. These findings are in line with another study that used primary healthcare data in the UK (OpenSAFELY platform). Tazare *et al*.^100^ revealed that the excess risk of major adverse CV events among previously hospitalized COVID-19 patients was similar to patients admitted with a diagnosis of pneumonia, although the risk of developing type 2 diabetes was higher after COVID-19.

### Prospective studies

In the post-acute period, cardiac abnormalities have been reported in several prospective observational studies.^101–134^ A summary of selected studies (*n* > 50) employing three widely used investigative tools—echocardiography, cardiac magnetic resonance (CMR), and cardiopulmonary exercise test—is provided in *Table 1* ^101,102,109,112,114–116,120–122,124,125,128,130,131,133,135^ and highlights the vast heterogeneity in the prevalence of abnormalities.

**Table 1:** Prevalence of cardiac abnormalities in studies (*n* > 50) that utilized echocardiography, cardiac magnetic resonance, and cardiopulmonary exercise test during follow-up of COVID-19 patients

First author	No. of patients	Age	Patient characteristics	Follow-up time	Controls	Cardiopulmonary symptoms	Echo findings
Echocardiography							
Hall *et al*.^135^	200	55 ± 15 years; 62% male	Hospitalized patients; 27.5% mechanical ventilation	4–6 weeks post-discharge	–	18% new-onset/worsening of dypsnoea	14% had either newly diagnosed or previously present abnormalities
Sechi *et al*.^130^	105	57 ± 14 years; 53% male	Hospitalized; 26% mechanical ventilation	Median 41 days post-symptom onset	105 matched controls	5% chest pain, 5% dyspnoea, 7% fatigue	No cardiac abnormalities
Catena *et al*.^116^	105	57 ± 14 years; 53% male	Hospitalized patients; 26% mechanical ventilation	Median 41 days post-symptom onset	–	5% chest pain, 5% dyspnoea, 7% fatigue	No differences in cardiac function between troponin+ and troponin− COVID-19 patients
de Graaf *et al*.^131^	81	61 ± 13 years; 63% male	Hospitalized patient; 41% mechanical ventilation	6 weeks post-discharge	–	62% dyspnoea, 14% chest pain, 32% limited functional status	18% LV dysfunction, 10% RV dysfunction
Moody *et al*.^125^	79	57 ± 11 years; 74% males	Hospitalized patients; 80% mechanically ventilated	3 months post-discharge	–	–	9% LV dysfunction, 14% RV dysfunction, 3% dilated LV, 9% dilated RV, 4% pericardial effusion
Sonnweber *et al*.^109^	145	57 ± 14 years; 57% males	75% hospitalized; 22% ICU admission	60 days and 100 days post-symptom onset	–	36% dyspnoea	3% LV systolic dysfunction—60 and 100 days, 55% diastolic dysfunction—60 days, 60% diastolic dysfunction—100 days, 10% pulmonary hypertension—60 and 100 days. Pericardial effusion 6% at 60 days and 1% at 100 days
CMR							
Kotecha *et al*.^101^	148	64 ± 12 years; 70% male	Severe COVID-19 and elevated troponin; 32% mechanically ventilated	Median 68 days post-discharge or confirmed diagnosis	40 co-morbidity matched and 40 healthy	No symptoms	11% LV dysfunction, 26% myocarditis, 23% ischaemia/infarction, 6% had dual pathology
Puntmann *et al*.^122^	100	49 ± 14 years; 53% male	67% non-hospitalized	Median 71 days post-positive COVID-19 test	50 healthy and 57 co-morbidity matched controls	36% breathlessness, 17% chest pain, 20% palpitations	60% myocardial inflammation, 78% any abnormality including LV, RV dysfunction, late gadolinium enhancement, and pericardial enhancement
Raman *et al*.^120^	58	55 ± 13 years; 59% male	Hospitalized patients; 21% mechanically ventilated	2–3 months post-symptom onset	30 co-morbidity matched controls	89% cardiopulmonary symptoms	No evidence of active myocardial oedema, no significant difference in scar burden with controls. Native T1 was elevated in 26%
Dennis *et al*.^121^	201	45 (21–71 years); 29% male	19% hospitalized	Median 141 day post-symptom onset	36 healthy controls	98% fatigue, 88% breathlessness, 76% chest pain	9% systolic dysfunction, 19% myocarditis
Zhou *et al*.^112^	97	47 ± 19 years; 54% male	Hospitalized patients (non-ventilated)	2–4 weeks after discharge	–	–	All patients had echo. 1% LV dysfunction. CMR in four patients. One had subepicardial hyper-enhancement with no elevated T2
Joy *et al*.^128^	74	39 (30–48 years); 38% male	Healthcare workers with predominantly mild infection; 3% hospitalized	6 months post-infection	75 SARS-CoV-2 antibody negative healthcare workers	11% symptomatic, 3% sore throat, 3% fatigue, 2% breathlessness	4% myocarditis like scar
Knight *et al*.^114^	29	64 ± 9 years; 83% male	Hospitalized with elevated troponin, 34% mechanically ventilated	Mean 46 days post-symptom onset	–	–	69% had pathology, 3% mild LV dysfunction, 3% severe biventricular dysfunction, 38% non-ischaemic injury, 17% ischaemic injury, 14% dual pathology, 7% pericardial effusion
Eiros *et al*.^124^	139	52 (41–57 years); 28% male	Healthcare workers; 16% hospitalized	Median 10.4 weeks post-symptom onset	–	27% fatigue,19% chest pain, 14% palpitations	75% had CMR abnormalities, 4% oedema on T2, 42% T1, 37% extracellular volume, 30% pericardial effusion, 5% LV dysfunction, 14% had pericarditis, 37% had myocarditis, 11% fulfilled criteria for both pericarditis and myocarditis
Myhre *et al*.^133^	58	56 (50–70 years); 56% male	Hospitalized; 19% mechanically ventilated	Median 175 days	32 healthy controls	64% fatigue, 55% dyspnoea, 4% chest pain	21% had pathology on CMR, 5% LV dysfunction, 17% late gadolinium enhancement
CPET							
Clavario *et al*.^102^	110	62 (54–69 years); 59% male	Hospitalized (excluded pts requiring mechanical ventilation/ICU)	3 months post-hospital discharge	–	74% at least one symptom. 50% dyspnoea, 26% chest pain, 49% fatigue, 23% palpitations	Median predicted pVO_2_ 90.9 (79.2–109). 35% had pVO_2_ < 80% predicted. DLE maximal strength independently associated with pVO_2_. 24% had cardiac limitation to exercise, 8% respiratory and cardiac, 47% non-cardiopulmonary limitation
Rinaldo *et al*.^115^	75	Mean 57 years; 57% males	Hospitalized (39 critical, 18 severe, 18 mild–moderate disease)	Mean 97 days from discharge	–	52% had dyspnoea with normal activity	Mean pVO_2_ 83% of predicted. 55% pVO_2_ < 85% of predicted. VE/VCO_2_ slope 28 ± 13. Patients with reduced exercise capacity had normal breathing reserve, 17% had circulatory limitation (heart rate reserve <15%), 20% reduced AT (<45%). Patients with a reduced exercise capacity showed an early AT, indicating a higher degree of deconditioning, lower peak oxygen pulse, reduced VO_2_/WR slope
Raman *et al*.^120^	58	55 ± 13 years; 59% male	Hospitalized patients at 3 months from symptom onset	3 months from symptom onset	30 co-morbidity matched controls	83% had at least one cardiopulmonary symptom	55% had pVO_2_ < 80% predicted, VE/VCO_2_ slope 33.^29–40^ HRR in first minute was slower in patients compared with controls

Data are presented as mean ± standard deviation or median (interquartile range).

AT, anaerobic threshold; BMI, body mass index; COVID, coronavirus disease; CMR, cardiac magnetic resonance; CPET, cardiopulmonary exercise test; DLco, carbon monoxide gas transfer; GLS, global longitudinal strain; HRR, heart rate recovery; ICU, intensive care unit; LV, left ventricle; PCR, polymerase chain reaction; pVO_2_, peak oxygen consumption; RV, right ventricle; PAP, pulmonary artery pressure; SARS-CoV-2, severe acute respiratory syndrome coronavirus 2; VE/VCO_2_, slope ventilatory equivalent for carbon dioxide; WR, work rate.

Numerous studies have evaluated the role of 12-lead electrocardiogram (ECG) in screening patients for post-acute cardiac manifestations,^36,103,117,124,136^ although commonly pre-COVID-19 control ECGs are missing. Dynamic ECG changes (e.g. depolarization, repolarization abnormalities, arrhythmias)^137,138^ while frequent during acute illness, tend to resolve in the majority of hospitalized patient by 6 months post-acute COVID-19 and are often comparable to risk-factor matched controls.^36,103,117,136^ Nevertheless, sinus arrhythmia is frequent in the post-acute phase and manifests as transient or sustained periods of sinus tachycardia or bradycardia.^103–105^ In a study of 234 patients, Radin *et al.* observed that 13.7% of patients wearing a Fitbit displayed persistently elevated heart rate (>5 beats above the pre-COVID baseline resting heart rate) up to 133 days post-infection.^139^ Currently, there are no published studies on the role of prolonged ECG monitoring (Holter) in post-COVID-19 management. Prior studies of post-influenza patients have demonstrated a high burden of atrial^140^ and ventricular arrhythmias, which are known to correlate with inflammatory markers.^141–145^ These findings imply that a subset of COVID-19 patients (e.g. with ongoing inflammation) may potentially benefit from ECG monitoring in the long term.

Both transthoracic echocardiography and CMR are cornerstones in the diagnosis of acute and chronic cardiac pathology.^146,147^ Cardiac abnormalities commonly reported on follow-up imaging include myopericarditis, right ventricular dysfunction, and ischaemia/infarction.

Myopericarditis may be suspected clinically based on the presence of one clinical (pericarditic chest pain, heart failure or progression of heart failure symptoms, palpitations, syncope, new-onset arrhythmia) and one diagnostic criteria (ECG abnormalities, troponin elevation, wall motion abnormalities on echocardiogram, CMR abnormalities) as per the 2013 European Society of Cardiology (ESC) Position statement^148^ and 2020 AHA scientific statement.^149,150^ While endomyocardial biopsy (EMB) is the gold standard investigation for histological evaluation of suspected fulminant cases,^148–150^ CMR remains the best alternative for non-invasive evaluation of stable cases^149,151^ and provides information on several pathological processes including myocardial oedema, hyperaemia, necrosis, and fibrosis by exploiting alterations in fundamental magnetic properties of the tissue (T1 and T2 relaxation). In 2018, the Journal of American College of Cardiology scientific expert panel provided recommendations for updated CMR criteria to improve detection of active myocarditis.^151^ This required an increase in at least one T1-based method including T1 mapping (sensitive to hyperaemia, fibrosis, necrosis, and oedema) and one T2-based method including T2 mapping (sensitive to oedema).^151^ Although the incorporation of quantitative tissue mapping has augmented the sensitivity of CMR for myocarditis, two limitations exist with such an approach. The first is that CMR diagnostic performance may be impacted by co-morbidities.^152,153^ The second is that readouts of T1 and T2 values of the myocardium are poorly standardized.^154^ As a result, combining quantitative CMR data from multiple centres is problematic.

In June 2020, a follow-up CMR study of 100 patients (67% non-hospitalized) reported an alarmingly high rate (60%) of persistent myocardial inflammation at 71 days post-infection.^122^ At least 22% of patients in this study were found to be co-morbid. Other studies have also reported a high prevalence of CMR abnormalities, though comparator groups were typically healthy making it challenging to rule out confounding effects of co-morbidities such as hypertension and diabetes. In contrast to these early studies, a subsequent study^128^ of healthcare workers, which enrolled co-morbidity matched controls with mild infection, reported a lower prevalence of CMR abnormalities, with no significant difference in 6-month CMR tissue abnormalities between seropositive and seronegative healthcare workers. Similarly, a study of 1285 UK Biobank participants^155^ with pre- and post-SARS-CoV-2 infection imaging revealed no link between prior infection and longitudinal changes in cardiac or aortic phenotypes before and after adjusting for potential confounders including co-morbidities.

There are now abundant studies evaluating the burden of myocarditis among athletes in view of associated risks of sudden cardiac death. The majority was undertaken within 1–2 months of infection^105,126,127,129^ and the prevalence of myocarditis on objective testing was found to be generally low (0–3%). Studies evaluating persistent symptom burden among athletes beyond 8 weeks of infection are lacking, though it is likely, given the lower risk profile of athletes (e.g. less likely to be obese and co-morbid), that ongoing symptoms are infrequent in this population.

The role of CMR in elucidating a cause for elevated troponin following acute COVID-19 is less contentious. Several prior studies^156–158^ have confirmed its incremental value in clarifying a diagnosis when the aetiology of troponin elevation is unclear. In a study of 148 troponin-positive patients, Kotecha *et al.* ^101^ reported that 26% had myocarditis, 22% had inducible ischaemia/infarction, and 6% had evidence of both.

Echocardiography is vital in the early diagnosis of cardiac pathology in COVID-19 infection (suspected myocarditis, Takotsubo syndrome, myocardial infarction, pericardial effusion, etc.), particularly where haemodynamic stability is uncertain.^69,159^ Right ventricular dilatation and dysfunction are the most common echocardiographic abnormalities with prognostic significance.^125,160,161^ When the acute infection abates,^161^ right ventricular abnormalities improve in most patients.^36,125^ Left ventricular systolic dysfunction is comparatively less frequent.^101,125^ Follow-up echo and CMR studies have confirmed that even among those with severe acute infection^125^ or elevated troponin,^101^ systolic impairment is rare, affecting up to 9–11% of patients. Concordant with this, in the UK-wide national follow-up study (PHOSP-COVID), levels of N-terminal pro B-type natriuretic peptide (NT-proBNP) were abnormal in only 7% of patients at 5 months post-hospitalization.^19^ Unlike systolic dysfunction, abnormalities in diastology may be common (up to 60% of hospitalized patients).^109^ The lack of pre-COVID imaging, however, makes it challenging to disaggregate what is cause and effect in patients.

Computed tomography (CT) angiography (CTA) has garnered considerable attention for its ability to detect pulmonary emboli,^162–164^ epicardial coronary stenoses, or vascular pathology (e.g. mural thrombus or vasculitis) related to acute SARS-CoV-2 infection. Perivascular fat attenuation index,^165,166^ a biomarker of vascular inflammation on CTA, has shown promise for prognostic risk stratification. In an early study by Kotanidis *et al*.,^167^ a new radiotranscriptomic signature of vascular inflammation demonstrated an association between SARS-CoV-2 variant B1.1.7, vascular inflammation, and increased mortality risk. Delayed arterial and venous thrombo-embolic complications^168–170^ have also been reported in the post-acute period. Dual-energy CTA, in a study of 55 patients at 3 months post-infection, detected both proximal arterial thrombosis (5.4%) and distal microangiopathy (65.5%) in a significant proportion of symptomatic patients.^171^

Cardiopulmonary exercise testing has shed light on the relevant pathophysiological brakes applied by COVID-19 on exercise capacity.^36,102,106–108,115,118–120^ Several studies have demonstrated a reduction in peak oxygen consumption post-acute COVID-19.^36,102,106,115,120,172^ The predominant mechanism for this finding seems to be muscular deficiencies (impairment in oxygen extraction^173^), manifesting primarily as submaximal exercise tests or an early anaerobic threshold. Generalized muscle wasting or sarcopenia is also common.^174,175^ Physical inactivity, cytokine storm, poor nutrition, intensive care therapy, mechanical ventilation, and drugs (e.g. dexamethasone) have all been implicated. The ratio of exercise minute ventilation coupled with carbon dioxide output (VE/VCO_2_), a marker of ventilatory efficiency, may also be abnormal.^36,102,120^ However, breathing reserve is relatively preserved, arguing against pulmonary factors and supporting hyperventilation or dysfunctional breathing as a potential cause.^172^ Heart rate recovery provides a surrogate measure of autonomic health in patients. Following COVID-19, delayed heart rate recovery has been noted in some patients,^36,176^ though the majority recovers spontaneously over time.^36^

There are now numerous reports^177–181^ and cohort studies^38^ where POTS has been suspected among patients. In a retrospective study of 20 patients^182^ referred to a dysautonomia clinic, orthostatic instability on tilt table test or 10 min stand test was observed in 75% of patients. Postural orthostatic tachycardia syndrome was the most common diagnosis in this study, followed by neurocardiogenic syncope (15%) and orthostatic hypotension (10%).

A table summarizing all the relevant cardiac investigations, their advantages, and role in post-COVID management can be found in the Supplementary material online, *Table S1*.

## Cardiovascular injury in the context of multiorgan involvement

Understanding cardiac involvement in the context of multisystem health can provide clues into mechanisms of ongoing injury through recognition of unique patterns of tissue damage (e.g. inflammatory changes or embolic manifestations). In an early study of 58 post-hospitalized COVID-19 patients and 30 matched controls, Raman *et al*.^120^ undertook multiorgan magnetic resonance imaging (MRI) and reported tissue abnormalities involving the lungs (60%), heart (26%), liver (10%), kidneys (29%), and brain (11%) in patients. Magnetic resonance imaging abnormalities in almost every organ correlated with inflammatory markers, suggesting that chronic inflammation could impede recovery. Following on from this work, the PHOSP-COVID study also demonstrated that failure to recover from multiorgan symptoms was associated with markers of persistent inflammation.^19^ In another study of 201 patients, Dennis *et al.* ^121^ evaluated the prevalence of multiorgan damage among predominantly non-hospitalized patients and noted that symptoms of long COVID clustered among those with multiorgan injury on MRI.

Persistent endothelial dysfunction,^47,183,184^ microvascular dysfunction,^101^ and prothrombotic tendencies^185^ may also contribute to multiorgan dysfunction.^186^ Selected studies of advanced imaging modalities including positron emission tomography, CT, and MRI have noted perfusion deficits in the heart and lungs^101,187,188^ of COVID-19 survivors at ∼40–60 days from infection. In one study of hospitalized patients, multiorgan MRI demonstrated evidence of small vessel disease (9.3%) and ischaemic changes (3.7%) in the brain (9.3%) and 1.9% had myocardial infarction^120^ 2–3 months post-infection. Another study of 104 hospitalized^189^ patients observed that inducible myocardial perfusion defects were common among patients with moderate to severe disease but did not differ in burden compared with co-morbidity and risk-factor matched controls. Several studies are currently underway to characterize the burden of vascular and thrombotic complications^190,191^ and to examine the potential benefits of prolonged antithrombotic (extended thromboprophylaxis) and vascular protective therapies (e.g. statins, risk-factor management) in post-acute COVID-19 patients as indicated in *Table 2* and Supplementary material online, *Table S2*.

**Table 2:** Examples of prospective clinical trials evaluating cardiovascular outcomes beyond 4 weeks of COVID-19

	Category	Title	Interventions	Outcome measures	Sponsor/collaborators	Enrolment	Funded by
NCT04324463	Cardiovascular	Anti-Coronavirus Therapies to Prevent Progression of Coronavirus Disease 2019 (COVID-19) Trial (ACTCOVID-19)	Colchicine Aspirin Rivaroxaban	Colchicine v control: 45 days composite of hospitalization and death, disease progression, composite of MACE Aspirin vs. control: 45 days composite of hospitalization and death, disease progression, composite of MACE Aspirin and rivaroxaban vs. control: 45 days composite of hospitalization and death, disease progression, composite of MACE	Population Health Research Institute, Bayer	4000	
NCT04381936	Cardiovascular	Randomized Evaluation of COVID-19 Therapy (RECOVERY)	Aspirin Colchicine Empagliflozin Anakinra Steroids	Primary and secondary outcome measures are 28-day mortality, need for ventilation, and hospital stay. Other outcome measures including risk of thrombotic event up to 6 months after randomization.	University of Oxford UK Research and Innovation, National Institute for Health Research, UK Wellcome, Bill and Melinda Gates Foundation, Department for International Development, UK, Health Data Research, UK Medical Research Council, Population Health Research Unit NIHR Clinical Trials Unit Support Funding, NIHR Health Protection Research Unit in Emerging and Zoonotic Infections	45 000	UK
NCT04662684	Cardiovascular	Medically Ill Hospitalized Patients for COVID-19 THrombosis Extended ProphyLaxis with Rivaroxaban ThErapy: The MICHELLE Trial (MICHELLE)	Rivaroxaban	Primary outcome measures are 35 days post-hospital discharge VTE and VTE-related death. Secondary outcome measures are 35 days post-hospital discharge bleeding and other outcome measures which are composite of myocardial infarction, stroke, arrhythmia, heart failure and all-cause death.	Science Valley Research Institute Bayer	320	Brazil
NCT04406389	Cardiovascular	Anticoagulation in Critically Ill Patients With COVID-19 (The IMPACT Trial) (IMPACT)	Enoxaparin Unfractionated heparin Fondaparinux Argatroban	Primary outcome measure 30-day mortality, secondary outcome measures include 6-month incidence of VTE, length of ICU stay, number of major bleeding events	Weill Medical College of Cornell University	186	USA
NCT04486508	Cardiovascular	Intermediate-dose vs. Standard Prophylactic Anticoagulation and Statin vs. Placebo in ICU Patients With COVID-19 (INSPIRATION)	Enoxaparin Unfractionated heparin (intermediate-dose) Atorvastatin Matched placebo	Primary outcome measure is 30 days composite of acute VTE, arterial thrombosis, mortality, treatment with ECMO Secondary outcome measures include 30-day MACE, arrhythmia, mortality, major bleeding, thrombocytopenia, raised liver enzymes, atrial fibrillation; 60, 90 days post-COVID-19 functional status	Rajaie Cardiovascular Medical and Research Center, Brigham and Women's Hospital, Tehran Heart Center, Masih Daneshvari Hospital Hazrat Rasool Hospital,Modarres Hospital, Firuzgar hospital affiliated to Iran University of Medical Sciences, Imam Khomeini Hospital Sina Hospital, Iran, Tabriz University of Medical Sciences Shariati Hospital, Imam Ali Hospital Labbafinejhad Hospital	600	Iran
NCT04900155	Cardiovascular	Evaluation of the effect of long-term lipid-lowering therapy in STEMI patients with Coronavirus Infection COVID-19 (CONTRAST-3)	Atorvastatin Atorvastatin and ezetimibe	Primary outcome measure is 96-week lipid profile, ventricular rhythm disturbance, electrical instability and autonomic regulation, left ventricular systolic function, myocardial deformation, MACE	Penza State University	200	Russia
NCT04460651	Cardiovascular	Prevention and Treatment of COVID-19 With EPA in Subjects at Risk-Intervention Trial (PREPARE-IT)	Icosapent ethyl Placebo	Primary outcome measure 60-day SARS-CoV-2 positivity, COVID-19 hospitalization; secondary outcomes measures include 60-day CRP, triglycerides, COVID-19-related hospitalization, 28-day non-fatal myocardial infarction and stroke	Estudios Clínicos Latino América Amarin Pharma Inc.	4093	Argentina
NCT04505098	Cardiovascular	A Pragmatic Randomized Trial of Icosapent Ethyl for High-Cardiovascular Risk Adults (MITIGATE)	Icosapent ethyl	Primary outcome measure is percentage of people with moderate to severe viral upper respiratory tract infection, worse clinical status. Secondary outcome measures included 12-month mortality, MACE, heart failure	Kaiser Permanente Amarin Corporation	16 500	California
NCT04350593	Cardiovascular	Dapagliflozin in Respiratory Failure in Patients With COVID-19 (DARE-19)	Dapagliflozin Placebo	Primary outcome measure 30-day organ dysfunction, heart failure, respiratory decompensation, ventricular tachycardia, vasopressor therapy, renal replacement therapy. Secondary outcome measure 30 days time to hospital discharge, days alive and free from respiratory decompensation	Saint Luke's Health System Saint Luke's Hospital of Kansas City AstraZeneca George Clinical Pty Ltd	1250	USA
ISRCTN11721294	Cardiac	Rehabilitation for cardiac arrhythmia	Rehabilitation	Primary outcome measure: autonomic function measured using 12-lead ECG Holter device to record the heart activity for 10 min and 24 h at baseline and after rehabilitation programme (6 weeks)	Saudi Arabian	110	Saudi Arabian Cultural Bureau (SACB)

COVID-19, coronavirus disease 2019; CRP, C-reactive protein; ECG, electrocardiogram; ECMO, extracorporeal membrane oxygenation; MACE, major adverse cardiovascular events; NYHA, New York Heart Association; SARS-CoV-2, severe acute respiratory syndrome coronavirus-2; VTE, venous thrombo-embolism; STEMI, ST elevation myocardial infarction.

## Chronic cardiovascular disease and impact of COVID-19

Up to a third of patients hospitalized with COVID-19 have a history of chronic CV diseases.^192,193^ Presence of concurrent cardiac disease is typically associated with higher in-hospital mortality, thrombo-embolic risk, and septic shock rates.^97,194^ Even in the post-acute period, patients with a history of heart failure are at a two- to four-fold risk of decompensation and mortality.^195,196^ Increased rates of heart failure exacerbation may present even beyond 30 days after SARS-CoV2 infection.^98^

One reason for the rising epidemic of post-discharge heart failure events is the withdrawal of guideline-directed medical therapy during acute illness.^197^ Investigators of the TRED-HF study^198^ have previously demonstrated the negative impact of heart failure therapy withdrawal in recovered dilated cardiomyopathy patients, resulting in relapse and poor outcomes. The successful resumption and optimization of heart failure therapies^199^ may therefore be important in halting heart failure readmissions post-acute COVID-19.

The shared cardiometabolic profile of COVID-19 and cardiac diseases implies that COVID-19 may play a role in destabilizing subclinical diseases (e.g. coronary artery disease and heart failure). This would explain the high prevalence of type 2 myocardial infarction^200^ in those with severe COVID-19 and also the rising incidence of ‘new’ CV diagnoses.^7,98,99^ Other mechanisms that may contribute include dysregulation of the renin–angiotensin–aldosterone system,^6^ endothelial dysfunction,^186,201,202^ renal injury,^203^ and steroid use.^204,205^

## Proposed model for investigation and management of cardiovascular sequelae and long COVID

**Figure 3:**
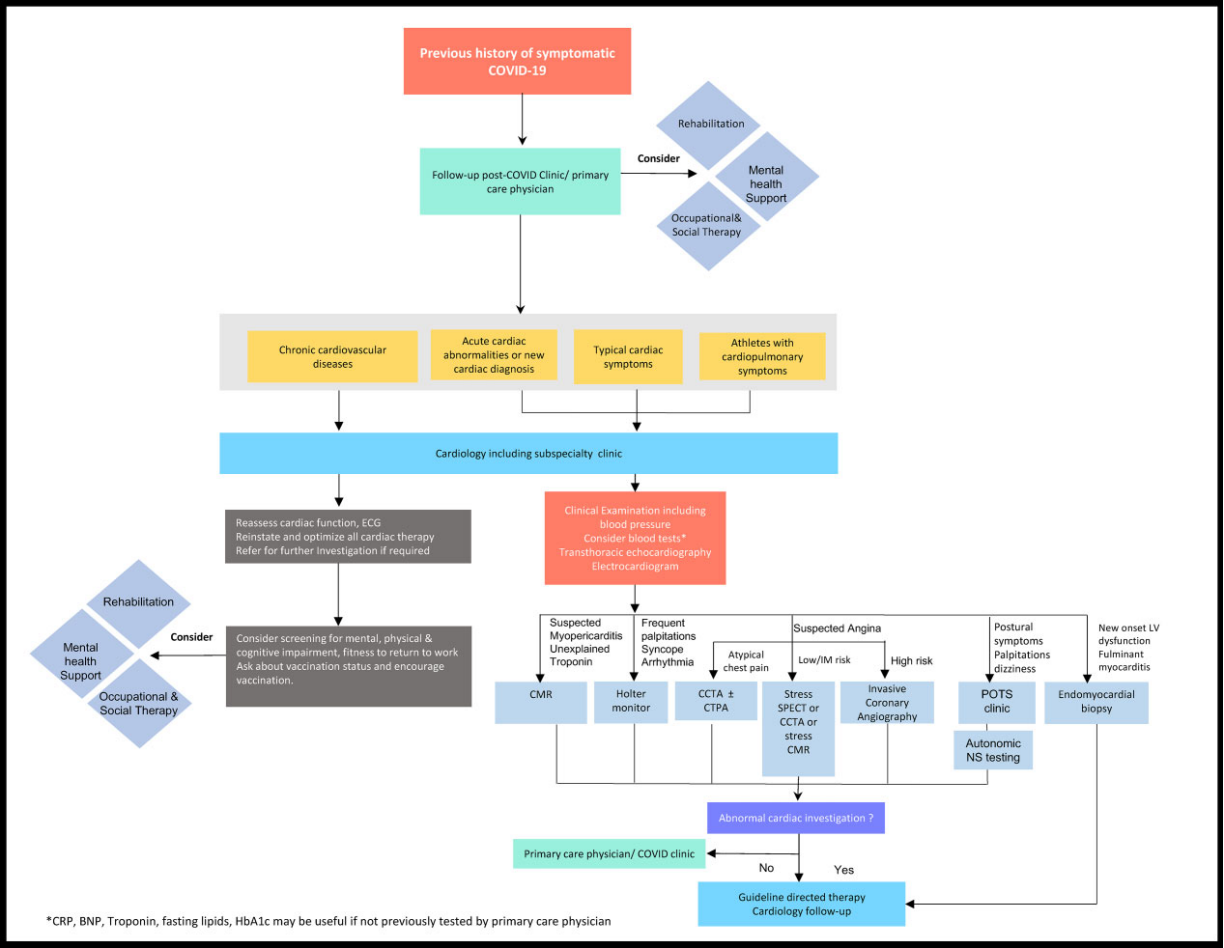
Suggested possible algorithm for follow-up care and management of post-acute cardiovascular sequelae of COVID-19. Given the pressures imposed by a backlog of referrals to cardiology services during the COVID-19 pandemic, appropriate referral of patients post-acute COVID-19 is of utmost importance. This figure provides a suggested framework for referral of long COVID patients to cardiology services and the potential role of additional investigations in specific cases where appropriate. BNP, B-type natriuretic peptide; CCTA, coronary computed tomography angiography; CMR, cardiac magnetic resonance; CRP, C-reactive protein; CTPA, computed tomography pulmonary angiography; ECG, electrocardiogram; HbA1c, glycated haemoglobin; NS, nervous system; POTS, postural orthostatic tachycardia syndrome; Trop, troponin.

Although the true burden of CV pathology post-acute COVID-19 remains elusive, the prevalence of cardiac symptoms in this phase appears high. There is a strong need for evidence in support of cost-effective strategies to exclude significant CV pathology. An approach considered reasonable by some experts^206–208^ involves screening of high-risk individuals for ongoing cardiac involvement including those with abnormal cardiac investigations during the acute phase, new CV diagnosis post-COVID-19, and athletes. *Figure 3* shows a possible pragmatic algorithm to guide physicians on the indication for cardiology follow-up and management approaches. Screening of high-risk individuals could comprise of a thorough history, clinical examination, blood test panel (C-reactive protein, troponin, B-type natriuretic peptide/NT-proBNP, glycated haemoglobin, lipids), ECG, and transthoracic echocardiography at least 8–12 weeks from infection. For patients with clinically significant abnormalities after the screening, additional testing is recommended. Non-invasive tests such as CMR, stress single positron emission computed tomography, Holter, coronary CTA can be considered following screening investigations; invasive coronary angiography or EMB may be indicated for high-risk individuals. Referral to specialist clinics (e.g. POTS, arrhythmia clinic, psychology support) should be considered where relevant. Patients with chronic CV diseases presenting for routine follow-up should be asked about their history of COVID-19 infection and vaccination status. A brief assessment of mental, physical, and cognitive health may be required for selected patients who report ongoing symptoms as this could facilitate early referral to appropriate services (rehabilitation,^209^ physiotherapy,^210^ psychology,^211^ occupational therapy, and social and welfare support) (*Figure 3*) and alleviate patient burden.

With regard to return-to-play guidance for athletes, numerous recommendations have been put forward by consensus societies.^212–214^ Although earlier guidelines adopted a conservative approach, recent studies of college and professional athletes have led to a revision^126,129^ in recommendations. Graded resumption to exercise and sports is now considered reasonable for mild infections, whereas exercise restriction for 3 months is still recommended for individuals with suspected myocarditis as per the 2019 position statement from the Sport Cardiology Section of the European Association of Preventive Cardiology.^215^

The management of COVID-19-related chronic myocarditis is a subject of considerable debate. In individuals with complicated (non-COVID) myocarditis (i.e. unexplained dilated left ventricle and severe dysfunction, ongoing troponin leak, advanced brady- or tachyarrhythmias), the ESC^148^ and AHA^149^ recommend EMB for clarification of myocarditis subtype to guide specific treatment options (e.g. immunomodulatory therapy vs. antivirals).^150^ Currently, there is no COVID-19 specific guidance on this, though several studies are underway to evaluate the most effective management strategy. The efficacy of oral non-steroidal anti-inflammatory drugs and/or colchicine is also being evaluated for COVID-19-associated pericarditis.^216^

For the management of post-COVID-19 acute coronary syndromes, patients are typically treated in accordance with the ESC^217^ and AHA^218^ guidelines released in 2020 and 2014, respectively. Similarly, heart failure management revolves around optimal utilization of contemporaneous therapies as per guidelines.^219,220^ There are currently no published trials on the efficacy of prolonged thromboprophylaxis post-acute COVID-19; however, numerous intervention trials (e.g. HEAL-COVID,^221^ STIMULATE ICP)^216^ are currently ongoing to address this gap.

After exclusion of significant CV and other organ pathology, the management of long COVID tends to be largely supportive.^222^ Given the strong association between obesity and long COVID, measures to reduce weight through caloric restriction, diet, tailored graded exercise, stress reduction, and good sleep hygiene could be beneficial in the long run,^223^ with growing evidence indicating its favourable effects on systemic inflammation,^224^ vascular dysfunction,^225^ and metabolic syndrome.^226^ Additionally, a pragmatic approach that is holistic and targeted at alleviating symptoms may also be required. Non-pharmacological approaches including pulmonary rehabilitation,^227^ breathing exercise,^228,229^ and alternative therapies^230^ (e.g. singing therapy,^231^ acupuncture, body rotation, and stretching) have also been suggested to help breathlessness symptoms. Those returning to work may benefit from phased return, allowing individuals with incomplete mental and physical recovery to gradually resume employment.^232^ Given that psychosocial factors are a major determinant of incomplete recovery,^38,233^ early referral for mental health assessment/cognitive behavioural therapy may benefit some patients.

Postural orthostatic tachycardia syndrome and symptoms of dysautonomia can be debilitating for patients.^178–181^ The management of POTS centres around accurate diagnosis following specialist assessment, correction of reversible causes (dehydration, heat), optimization of chronic disease management, and patient education. In some patients with ongoing palpitations, beta-blockers can be helpful in treating symptoms.^234^ Graded exercise programmes^235^ encouraging patients to adopt an upright posture may attenuate postural symptoms after prolonged bed rest. Compression panty-hose style stockings with 30–40 mmHg counter pressure may help symptoms of orthostatic hypotension through reduced peripheral venous pooling.^236^ In the event that symptoms persist despite compliance with the aforementioned approaches, pharmacological therapies (e.g. ivabradine, fludrocortisone, midodrine, clonidine, and methyldopa^237^) may be considered.

## Special considerations

### Impact of acute therapies

The vast majority of persistent CV abnormalities following COVID-19 is due to tissue injury sustained during the acute illness. The impact of acute therapies on long-term CV health deserves further investigation. Currently, anti-inflammatory drugs such as dexamethasone^238^ and tocilizumab^239^ or antivirals such as remdesivir^240^ have been identified as key weapons in the therapeutic armamentarium against severe COVID-19. However, the extent to which they affect cardiopulmonary recovery in the long term is still unclear and data regarding cardiac injury rates are not yet widely available. Whether or not ongoing inflammation in long COVID may reflect a rebound phenomenon in dexamethasone- or tocilizumab-treated patients also warrants further study. The complicated role of anticoagulation in patients deserves some consideration. In the acute phase, mounting evidence confirms a lack of benefit of aspirin in reducing mortality among hospitalized^241^ and non-hospitalized outpatients.^242^ Data in support of therapeutic dose anticoagulation are mixed with the severity of illness (non-critical hospitalized patients benefitting most) being a critical determinant of treatment success.^243^ The multiplatform adaptive randomized controlled clinical trial,^244^ which combined data from ACTIV-4a, REMAP-CAP, and ATTACC studies, reported an improved survival until hospital discharge and organ support-free days with therapeutic dose heparin in moderately ill patients but not during critical illness. In contrast, other studies (ACTION,^245^ INPIRATION,^246^ and RAPID trials)^247^ reported no difference in primary outcome measures among patients receiving therapeutic vs. prophylactic dose anticoagulation. Investigators of the ACTIV-4B^242^ study also reported no improvement in 45-day survival among non-hospitalized patients receiving aspirin, low dose, and high dose apixaban vs. placebo. Further research is therefore needed to better understand the long-term benefits of anticoagulation in patients.

### Role of vaccination—risks vs. benefits

The most effective way of preventing serious complications from SARS-CoV-2 infection is through vaccination.^248–254^ Previous experience with influenza vaccine has taught us to expect a favourable relationship between vaccination and CV outcomes.^255,256^ There are at least eight major SARS-CoV-2 vaccines available globally, with excellent efficacy. Early data from a patient-led observational study^257^ has hinted at the possibility of long COVID symptoms being alleviated through vaccination. Of 900 people with long COVID, 56.7% of those vaccinated saw an overall improvement, 18.7% a deterioration, and 24.6% were unchanged post-vaccination. In another survey study (COVID symptom app study),^258,259^ the odds of experiencing symptoms more than 28 days post-vaccination was halved by two vaccinations (*n* = 906). Some experts posit that an accelerated viral clearance and a muted chronic inflammatory response could explain symptom reduction following vaccination.^260^

While there is little doubt that early inoculation confers the greatest protection against severe COVID-19, rare cases of vaccine-induced adverse effects have led to a rise in vaccine hesitancy.^261^ In particular, two widely available vaccine strategies including mRNA vaccines^250,262^ and vector-based (ChAdOx1 nCov-19^263^ and Ad26.COV2.S/Janssen)^251^ have been linked to cases of myocarditis^264–266^ and vaccine-induced prothrombotic immune thrombocytopenia (VITT),^267^ respectively. Although rare (∼5 per million), VITT due to antibodies to platelet factor 4 typically occurs following a single vaccine dose and may be fatal in some (pulmonary embolus, cerebral venous thrombosis). In contrast, myocarditis, potentially due to an autoimmune response (triggered by molecular mimicry between spike protein and self-antigen), tends to be more common after the second mRNA vaccine dose but is comparatively less life-threatening as the majority of cases spontaneously resolves.^264–266^

## Future direction

Current evidence for the treatment of long COVID is lacking, though many clinical trials for long COVID and CV sequelae (*Table 2*) are currently underway. A list of selected clinical trials in long COVID are presented in Supplementary material online, *Table S2* to demonstrate the diversity of treatments under investigation. Studies include a variety of rehabilitation programmes (telemedicine and face–face) for treatment of fatigue, cognitive decline and breathlessness, therapies targeted at cognition (e.g. transcranial stimulation), metabolic modulators (e.g. niagen), immunomodulatory therapies (e.g. steroids, laranilubmab, tocilizumab, atorvastatin, colchicine), antifibrotic treatments (e.g. pirfenidone, LYT-100), and anticoagulation (e.g. apixaban). The World Health Organization (see https://clinicaltrials.gov/ct2/who_table or https://www.who.int/clinical-trials-registry-platform) and ClinicalTrials.gov (see https://clinicaltrials.gov/ct2/results?cond=COVID-19) list more than 730 studies related to COVID-19; >80 have a major emphasis on long-term CV outcomes. As examples, selected studies with 400 or more participants are listed in *Table 3*.

**Table 3:** Examples of large (*n* ≥ 400) prospective observational studies evaluating short- and long-term cardiovascular outcomes associated with COVID-19

National clinical trials number	Title	Cardiovascular outcome measures	Age	Enrolment	Study type
NCT04358029	Cardiac arrhythmias in patients with coronavirus disease (COVID-19)	Cardiac arrhythmias, mode of death, number of recurrence of atrial arrhythmias	Child, adult, older adult	10 000	Observational
NCT04465552	Arrhythmic manifestations and management in hospitalized COVID-19 patients	Arrhythmic manifestations employed treatment strategies and long-term outcomes in hospitalized COVID-19 patients	18 years and older (adult, older adult)	666	Observational
NCT04508712	Long-term outcomes in patients with COVID-19	Cardiac function	18 years and older (adult, older adult)	900	Observational
NCT04624503	Prognostic and clinical impact of cardiovascular involvement in patients With COVID-19 (CARDIO-COVID)	Cardiovascular mortality, all-cause mortality, major adverse cardiovascular events, NYHA class, left ventricular systolic function (cardiac magnetic resonance, echocardiography)	18–85 years (adult, older adult)	500	Observational
NCT04359927	Long-term effects of coronavirus disease 2019 on the cardiovascular system: CV COVID-19 Registry (CV-COVID-19)	Cardiovascular mortality, acute myocardial infarction, stroke	18 years and older (adult, older adult)	10 000	Observational
NCT04724707	Russian Cardiovascular Registry of COVID-19	Death, hospitalization for cardiovascular reasons, mechanical support or heart transplant, ICD or CRT, Arrythmias	18 years and older (adult, older adult)	900	Observational
NCT04384029	The Geneva COVID-19 CVD Study	Clinical outcomes related to pre-existing cardiovascular risk factors at admission, new onset of CVD induced by COVID-19	18 years and older (adult, older adult)	1927	Observational
NCT04375748	Hospital registry of acute myocarditis: evolution of the proportion of positive SARS-CoV-2 (COVID-19) cases (MYOCOVID)	Prognosis of the acute myocarditis, cardiac MRI parameters	Child, adult, older adult	400	Observational

COVID-19, coronavirus disease 2019; CRT, cardiac resynchronization therapy; CVD, cardiovascular disease; ICD, implantable cardioverter-defibrillator; MRI, magnetic resonance imaging; NYHA, New York Heart Association; ICD, implantable cardioverter defibrillator; SARS-CoV-2, severe acute respiratory syndrome coronavirus 2; VTE, venous thrombo-embolism.

## Research priorities

Current priorities for research include (i) establishing the prevalence of persistent or chronic SARS-CoV-2 induced CV injury; (ii) elucidating causal mechanisms including the role of immune system, obesity, endotheliopathy, and genetic predispositions; (iii) developing an understanding of CV symptom burden (as part of the long COVID spectrum) and its association with pathology; (iv) developing and refining scalable diagnostic methods with high specificity for COVID-19-associated CV complications (including POTS); (v) identifying novel therapeutic solutions or repurposing old drugs that can protect or reverse COVID-19-associated long-term CV injury; (vi) evaluating the role of vaccination and SARS-CoV-2 variants on cardiac injury; (vii) evaluating the long-term impact of SARS-CoV-2 infection on those with pre-existing cardiac diseases and future risk of heart failure, ischaemic events, and arrhythmias; (viii) evaluating the effects of COVID-19-related autonomic dysfunction on CV homeostasis; and (ix) understanding the impact of long COVID on healthcare costs and on working population.^4,230,233^ A concerning trend observed in recent studies^36,19,121,268^ is the marked dissociation seen between symptoms and objective measures of health highlighting the limitations of routine clinical investigations. In this regard, deeper phenotyping efforts including advanced cardiopulmonary imaging (e.g. hyperpolarized xenon^269^), mass spectrometry,^270^ metabolomics,^271^ proteomics,^272^ whole-genome sequencing, and gut microbiome^273^ studies promise to unscramble the Rubix cube of pathophysiological processes that underpin long COVID. It is anticipated that multiple endophenotypes^230,233^ (inflammatory, metabolic, autoimmune, neurocognitive, psychological^233^) will surface, through artificial intelligence-driven data analysis, enabling precision diagnostics, prognostic risk prediction, and therapeutics for patients.

## Conclusion

Long COVID is emerging as a major public health issue. Our current understanding of pathophysiological mechanisms and treatment options remains limited; however, there is great optimism as several national and international research initiatives promise to disentangle the complexities of this disease. The high burden of cardiopulmonary symptoms along with other organ manifestations underscores the need for multispecialty input,^274,275^ a model that is likely to also profit other chronic diseases. Proactive screening and investigation, where appropriate, could allay fears and anxiety among patients. Considerable efforts to find the right balance between cost-effective investigations and benefit to patients are needed to ensure sustainable service provision in these challenging economic times. Finally, the vast inequalities^43,276^ in healthcare provision exposed by COVID-19 will continue to be magnified by long COVID, a problem that calls for global humanitarian efforts to promote and fund equitable access to healthcare, social and welfare support, and vaccines across the world.

## Supplementary material

Supplementary material is available at *European Heart Journal* online.

## Supplementary Material

ehac031_Supplementary_Data
