# Supplementary material for: Long COVID: post-acute sequelae of COVID-19 with a cardiovascular focus
Source: Eur Heart J. 2022 Feb 18;43(11):1157–72. doi: 10.1093/eurheartj/ehac031 (PMC8903393; doi:10.1093/eurheartj/ehac031)
Supplement: ehac031_Supplementary_Data [file ehac031_supplementary_data.docx]

**Supplementary Table 1: Summary of all the relevant cardiac investigations, their advantages and role in post-COVID management**

|  | **Advantages** | **Role in post-COVID management** |
| --- | --- | --- |
| Echocardiography | Highly portable, non-ionizing, permits targeted assessment, enables accurate assessment of flow, validated against invasive measures of diastolic function | Evaluation of suspected myopericarditis, assessment of right ventricular function in the context of pulmonary emboli, screening for new onset biventricular dysfunction, atrial appendage for thrombus, Assessment of diastolic function |
| Magnetic Resonance Imaging | Accurate and reproducible for the assessment of biatrial and biventricular volumes, function and mass, permits tissue characterisation providing information on a range of histopathology (myocarditis versus infarction). Permits assessment of pulmonary vascular calibre and diastolic strain rate and allows for assessment of myocardial perfusion at rest and following vasodilator and exercise provocation. | Diagnosis of myopericarditis, myocardial infarction, cardiomyopathy, monitoring of disease progression and recovery of tissue abnormalities, assessment of biventricular function, assessment of micro and macrovascular function, assessment of diastolic function. |
| Cardiopulmonary exercise test | Permits quantification of aerobic capacity and allows assessment of the relationship between physiological response with symptom onset. Enables assessment of haemodynamics , ECG and gas exchange during exercise. | Objective assessment of exercise capacity and contributing factors to delineate the predominant cause (cardiac, pulmonary, skeletal muscle, anaemia) for exercise intolerance. Enables assessment autonomic response during recovery. |
| CT pulmonary and coronary angiography | Non-invasive evaluation of coronary artery and pulmonary artery, rapid acquisition, high negative and positive predictive value for disease | Detection of pulmonary and coronary thrombosis, vasculitis, perivascular inflammation and thrombus burden in large vessels. |
| Cardiac SPECT | Non-invasive evaluation of myocardial perfusion, enables visual/qualitative assessment or regions of ischaemia | Provides function information/haemodynamic significance of coronary stenosis (micro and macrovascular) |
| ECG monitor | Non-invasive evaluation of electrical rhythm and pathological electrical changes. | Helpful for the diagnosis of atrial and ventricular arrhythmia, orthostatic tachycardia syndrome and ischaemic ECG changes during exercise. |
| Tilt Table test | Evaluation of autonomic response | Useful in the diagnosis of POTS, orthostatic hypotension, neurogenic syncope, vasovagal syncope. |
| CT computed tomography; ECG electrocardiography; POTS Postural Orthostatic Tachycardia Syndrome; SPECT Single Photon Emission Computed Tomography. | | |

**Supplementary Table 2. Examples of prospective intervention studies in long COVID.**

|  | **Category** | **Title** | **Interventions** | **Outcome Measures** | **Sponsor/Collaborators** | **Enrollment** | **Funded By** |
| --- | --- | --- | --- | --- | --- | --- | --- |
| NCT04876417 | **Fatigue** | Transcranial direct current stimulation (tDCS) for Post COVID-19 Fatigue | Device: Transcranial direct current stimulation | Fatigue assessment scale (FAS), Fatigue Severity Scale (FSS), Fatigue testing of the knee muscles of both legs  6-minute walk test | University of Iowa | 50 | Other |
| NCT04950803 | **Fatigue** | A Randomised-Controlled Trial of an Oral Microbiome Immunity Formula in Recovered COVID-19 Patients | Drug: Microbiome immunity formula | Long COVID, readmission to hospital or multisystem complications, change in metabolic syndrome score, quality of life, health care utilization and hospital admission, blood immunity profile, faecal metabolites. | University of Hong Kong | 280 | Industry |
| NCT04924881 | **Fatigue** | Chinese Medicine for Patients With LCOVID-19 Symptoms | Drug: COVID Rehab Formula granules | Fatigue severity score, dyspnea evaluation, EuroQol 5 Dimension 5 Level (EQ-5D-5L), pulmonary function testing, adverse events | Chinese University of Hong Kong | 68 | Other |
| NCT04950673 | **Neurocognitive** | Open-label, Post-marketing, Prospective Study to Assess Impact of COVID-19 on Cognitive Function in Patients | Device: Cognivue | Cognitive assessment battery measures | Cognivue, Inc., University of Southern California, Case Western Reserve University, University of California, Irvine | 1000 | Industry |
| NCT04809974 | **Neurocognitive** | Clinical Trial of Niagen (nicotinamide riboside) to Examine Recovery in People With Persistent Cognitive and Physical Symptoms After COVID-19 Illness (long COVID) | Drug: Niagen | Executive functioning and memory composite scores, depression symptoms, anxiety symptoms, COVID-related physical symptoms | Massachusetts General Hospital | 100 | Other |
| NCT04950725 | **Physical Therapy/Rehab** | Covid-19 Virtual Recovery Study | Strength RMT and nasal breathing, endurance | Change in Phonation time, Sit to stand executions, number of reported COVID symptoms, breathing difficulty, physical activity, and reported cognition | Mayo Clinic | 1500 | Other |
| NCT04900961 | **Physical Therapy/Rehab** | CISCO-21 Prevent and Treat long COVID-19. | Resistance Exercise | Incremental Shuttle Walk Test, spirometry, handgrip strength, EQ-5D-5L, Duke Activity Status Index, Fatigue questionnaire, Fried Frailty phenotype | NHS Greater Glasgow and Clyde, University of Glasgow, Chief Scientist Office of the Scottish Government | 220 | Other |
| NCT04628039 | **Physical Therapy/Rehab** | Chronic Lung Disease and COVID-19: Understanding Severity, Recovery and Rehabilitation Needs | Rehabilitation-focused program | EQ-5D-5L visual analog score, WHO Disability Assessment Schedule 2.0 (WHODAS 2.0), overall utility index | VA Office of Research and Development | 506 | U.S. Fed |
| NCT04988282 | **Pulmonary** | Pirfenidone Compared to Placebo in Post-COVID19 Pulmonary Fibrosis COVID-19 (FIBRO-COVID) | Drug: Pirfenidone | Change from baseline in %FVC and change from baseline in % fibrosis on HRCT, functional improvement in FVC, change in exercise capacity, hospitalization, lung transplantation, death | University Hospital of Bellvitge | 148 | Spain |
| NCT04988282 | **Pulmonary** | Systemic Corticosteroids in Treatment of Post-COVID-19 Interstitial Lung Disease | Drug: Methylprednisolone | Radiological improvement, Improvement of diffusion capacity of lung for carbon monoxide (DLCO), Improvement of Forced Vital Capacity (FVC), Arterial oxygen saturation (SaO2), Improvement of Exercise Capacity | Turkish Thoracic Society | 642 | Other |
| NCT04695704 | **Pulmonary** | Study to assess the efficacy of montelukast in improving respiratory symptoms in patients with long COVID: E-SPERANZA COVID PROJECT | Drug: Montelukast | Quality of Life of respiratory symptoms according to COPD assessment test (CAT), 1min sit-tot-stand test, O_2_ desaturation, visual analogue scale, mortality, hospital readmission. | Institut Català de la Salut, Turkey | 284 | Other |
| NCT04801940 | **Multisystem** | HElping Alleviate the Longer-term Consequences of COVID-19 (HEAL-COVID) | Drugs Atorvastatin  Apixaban  Standard of Care | Hospital free survival over 12 months  Fatigue Assessment of Chronic Illness Therapy (FACIT)-fatigue scale, EQ-5D-5L, Patient health questionnaire (PHQ-9), Generalized Anxiety Disorder-7 scale  PTSD Checklist (PCL)-Civilian version  COVID-19 core outcome measure | National Institute of Health Research  University of Cambridge  University of Liverpool, United Kingdom | 2631 | UK Fed |
| N/A | **Multisystem** | Symptoms, Trajectory, Inequalities and Management: Understanding long COVID to Address and Transform Existing Integrated Care Pathways  (STIMULATE ICP) | Drugs: Colchicine  Aspirin  Standard of Care | N/A | National Institute of Health Research  University College London  United Kingdom | 4500 | UK Fed |
| NCT04842448 | **Multisystem** | Safety and Efficacy of Hyperbaric Oxygen Therapy for long COVID syndrome | Intervention: Hyperbaric oxygen therapy | Short Form Health Survey-36  Secondary: EQ-5D-5L, 6-minute walk test, endothelial function, 30/50 min chair stand | Karolinska University Hospital, Sweden | 80 | Other |
| CAT chronic obstructive pulmonary disease assessment test; COVID-19 Coronavirus disease; CRT cardiac resynchronization therapy; CVD cardiovascular disease; EuroQol 5 Dimension 5 Level (EQ-5D-5L); FAS fatigue assessment scale; FACIT fatigue assessment of chronic illness therapy; FSS fatigue severity scale; FVC forced vital capacity; HRCT high resolution computed tomography; NYHA new York heart association; ICD implantable cardioverter defibrillator; PTSD post-traumatic stress disorder; SARS-CoV-2 Severe acute respiratory syndrome coronavirus-2; Transcranial direct current stimulation tDCS. | | | | | | | |
